# Supplementary figures and images for: Spike Rate Inference from Mouse Spinal Cord Calcium Imaging Data
Source: J Neurosci. 2025 Mar 24;45(18):e1187242025. doi: 10.1523/JNEUROSCI.1187-24.2025 (PMC12044035; doi:10.1523/JNEUROSCI.1187-24.2025)

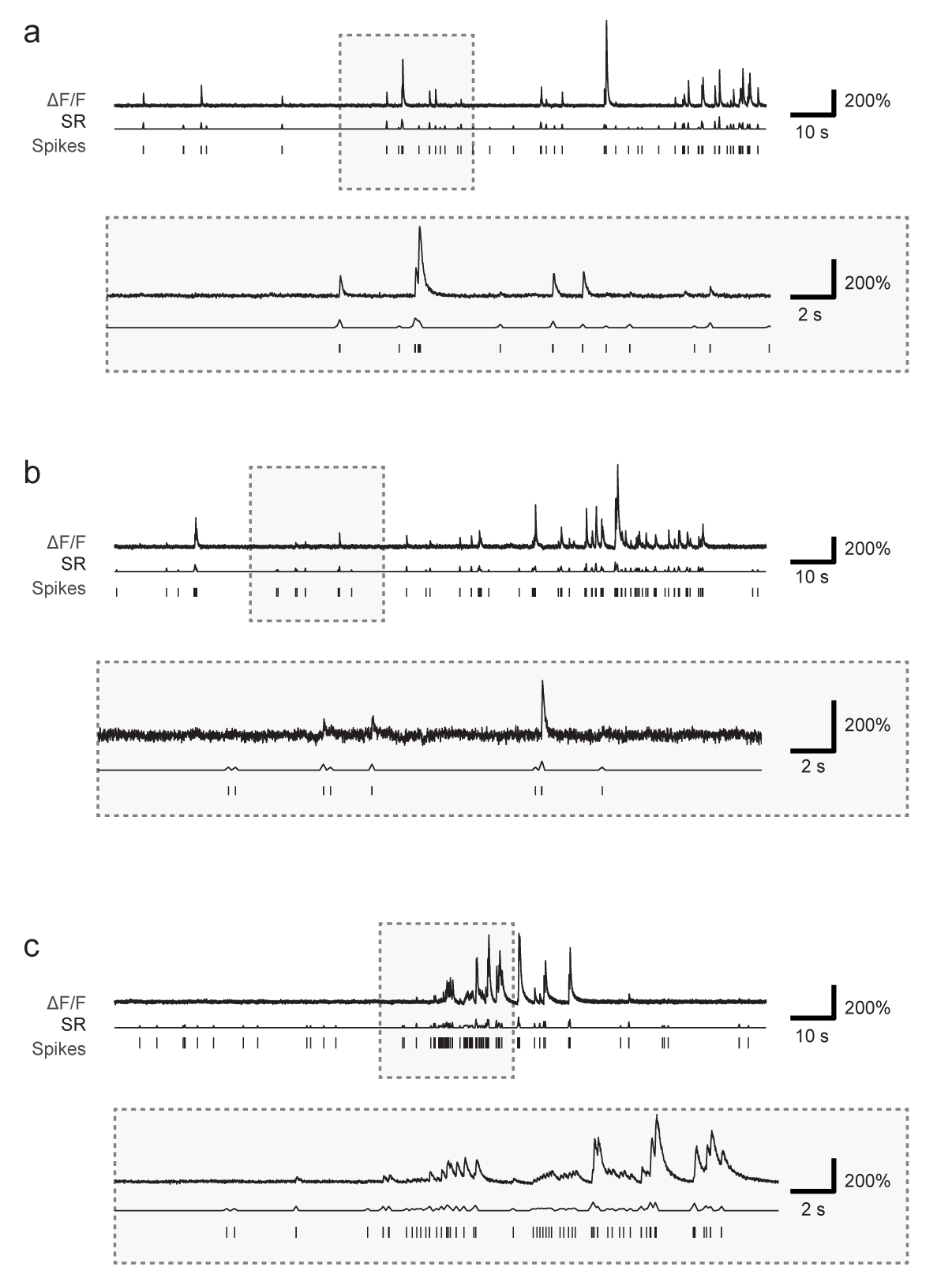

Supplement: Figure 1-1 — Simultaneous recording of action potentials and calcium signals in layer 2/3 pyramidal neurons from mouse cortex. Same presentation of example recordings as in Fig. 1e-j but for the ‘cortex dataset’, comprising ground truth recordings from four different transgenic mouse lines expressing GCaMP6f or GCaMP6 s in layer 2/3 neurons in mouse visual cortex. Recordings were performed in visual cortex of lightly anesthetized mice and included periods without and with visual stimulation using static and drifting gratings (Huang et al., 2021). ΔF/F, normalized fluorescence extracted from calcium imaging; SR, smoothed spike rate derived from electrophysiological spike times; Spikes, spike times detected from the electrophysiological recording. Download Figure 1-1, TIF file. [file jneuro-45-e1187242025-s001.tif]

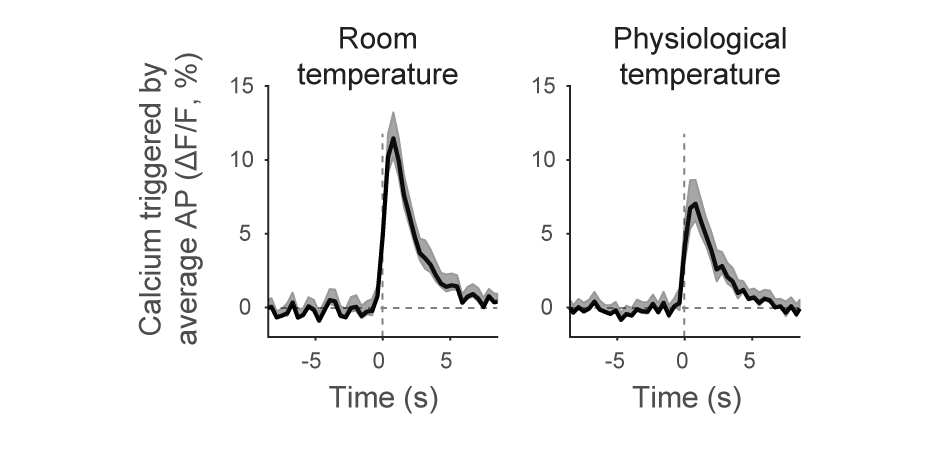

Supplement: Figure 3-1 — Temperature-dependence of the calcium transient evoked by the average spike. Calcium response (ΔF/F) for the average action potential across neurons, computed by linear deconvolution of the ground truth recording. Left: All neurons from the SC Glu+ dataset. Right: Recordings from the SC Glu+ dataset (11 out of 69 recordings in 5 out of 21 neurons) that were performed at physiological temperature (37°C). No slowing of indicator kinetics for room temperature is visible from this dataset (single exponential fit: τ = 2.9 ± 0.5 s for room temperature, 3.0 ± 0.6 s for physiological temperature; fit ± 90% confidence intervals). Download Figure 3-1, TIF file. [file jneuro-45-e1187242025-s002.tif]

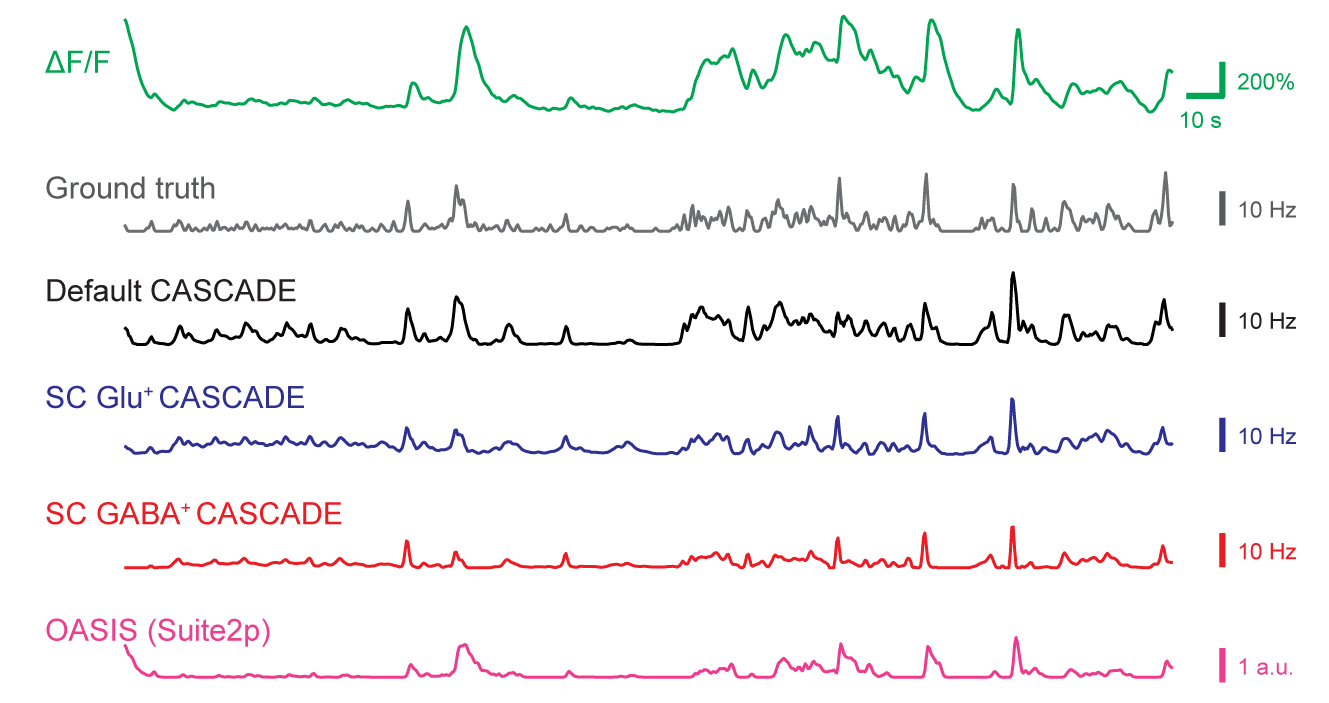

Supplement: Figure 4-1 — Example predictions for a lower sampling rate. Examples of an extracted ground truth recording (same as in Fig. 4a) together with the ground truth spike rate and spike rate predictions with CASCADE and OASIS. Sampled at 2.5 Hz with a standardized noise level of “7”. Download Figure 4-1, TIF file. [file jneuro-45-e1187242025-s003.tif]

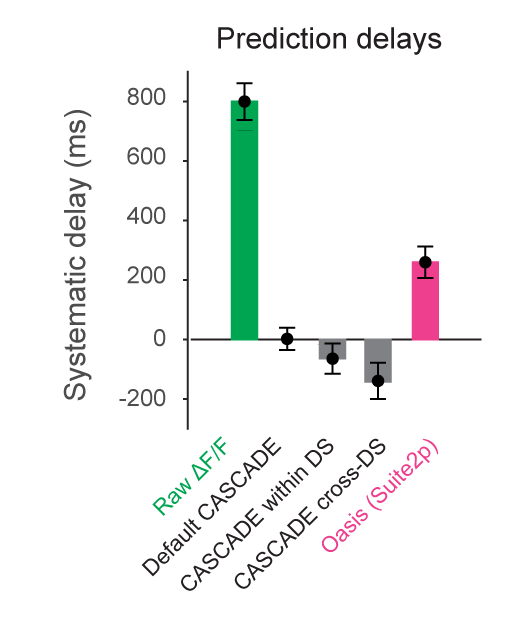

Supplement: Figure 4-2 — Systematic delay of spike rate inference with respect to ground truth. Quantified for the approaches described in Fig. 4a. “Within-DS” indicates that CASCADE was trained with matching datasets (e.g., CASCADE trained on glutamatergic neurons and applied to glutamatergic neurons), while “cross-DS” was trained with non-matching datasets. Consistent with previous analyses (Rupprecht et al., 2021), not only raw ΔF/F but also OASIS tended to result in a systematic delay of predictions compared to ground truth, as opposed to default or retrained CASCADE. All analyses in Fig. 4 to 7 are corrected for these systematic delays. Download Figure 4-2, TIF file. [file jneuro-45-e1187242025-s004.tif]

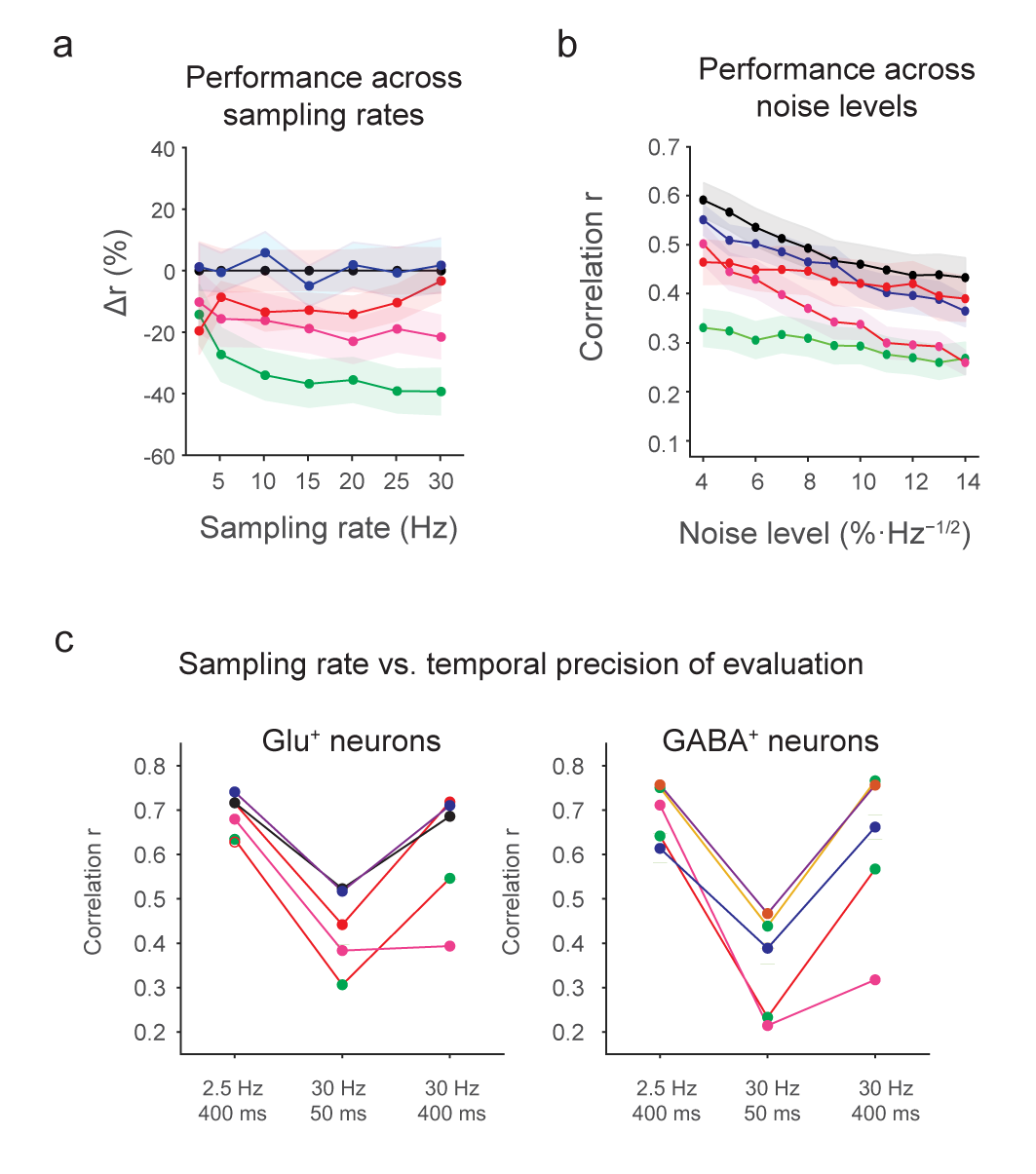

Supplement: Figure 4-3 — Further comparison of the performance of spike rate inference across algorithms. a, Quantification of performance for the glutamatergic spinal cord dataset across re-sampled imaging rates. The same quantification for the GABAergic spinal cord dataset was shown in Fig. 4 g. b, Quantification of performance for the glutamatergic spinal cord dataset across noise levels. The same quantification for the GABAergic spinal cord dataset was shown in Fig. 4 h. c, Control analysis to show that performance (“correlation”) is recovered for high imaging rates when the evaluation criterion (temporal smoothing applied before correlation with ground truth) is matched to low imaging rates. For example, the performance underlying the left-most datapoints were measured at an imaging rate of 2.5 Hz with a smoothing window of 400 ms. Therefore, spike rate inference reconstructs spike rates with similar accuracy from fast and slow imaging data when evaluated with the same slow temporal precision. Download Figure 4-3, TIF file. [file jneuro-45-e1187242025-s005.tif]

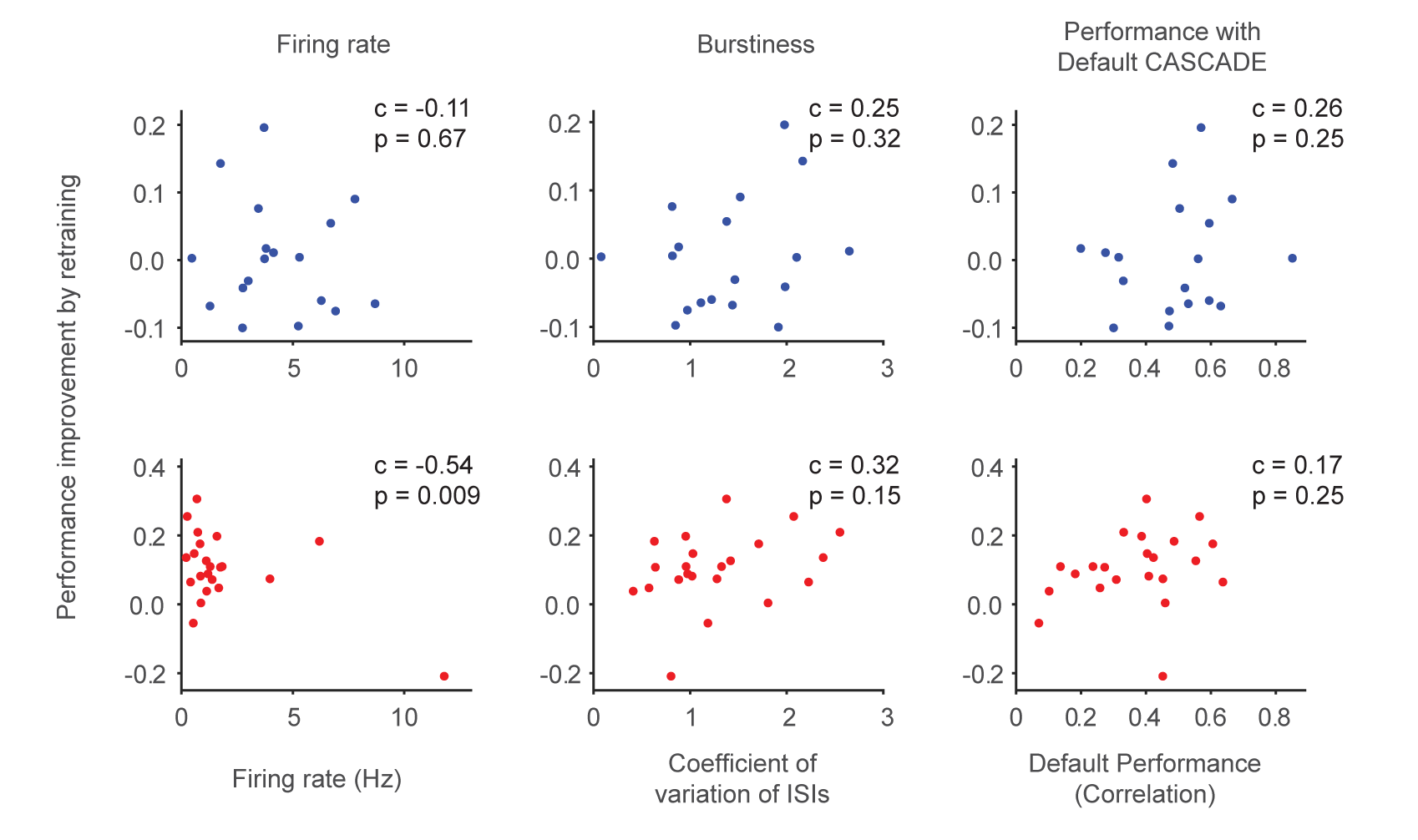

Supplement: Figure 4-4 — Dependence of performance improvement on cellular characteristics. To test whether specific subtypes of spinal cord dorsal horn neurons improved more than others with retraining, we explained the improvement by retraining (y-axis) by other variables obtained for each cell (average firing rate, burstiness, see Fig. 2; and the performance when applying Default CASCADE). Top row: glutamatergic neurons. Bottom row: GABAergic neurons. We did not find any significant correlation (p > 0.05; correlation values c and significance values p indicated in the figure) for either excitatory (blue) or inhibitory (red) spinal cord neurons. The relationship between firing rate and performance improvement was statistically significant for inhibitory neurons (p = 0.009), but this effect was driven by a single outlier (p = 0.90 after removal of the single outlier). As a conclusion, no cellular properties potentially indicative of cellular subtypes in the spinal cord were found that were predictive of performance improvement after retraining. It is therefore reasonable to assume that, within the limitations of these limited datasets, performance improvements upon retraining of CASCADE affected most or all neurons without a specific pattern. Download Figure 4-4, TIF file. [file jneuro-45-e1187242025-s006.tif]

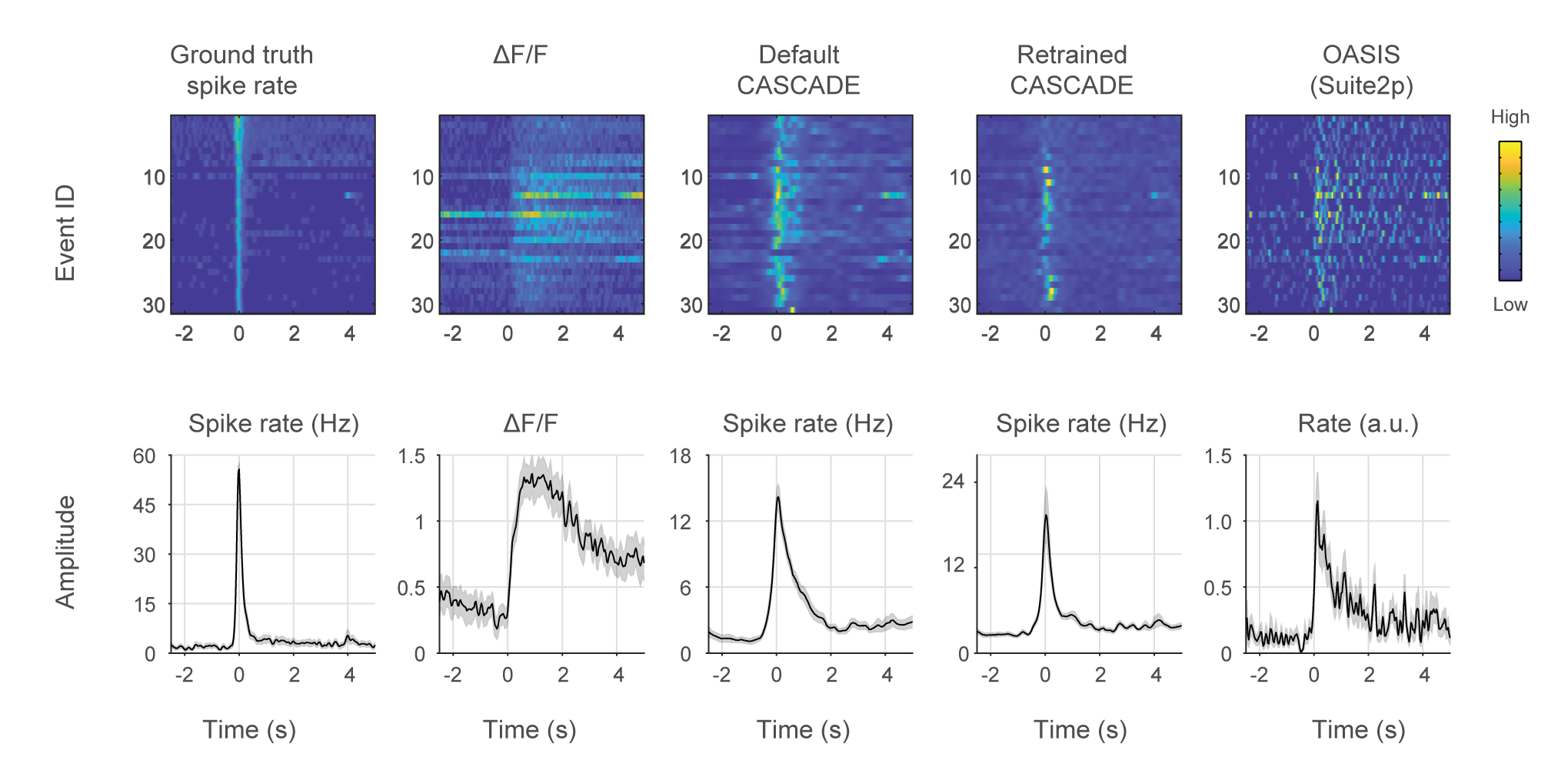

Supplement: Figure 5-1 — Spike rate inference for high-frequency spike events for GABAergic neurons in spinal cord. Top row: High-frequency spike events for the GABAergic neuron dataset, with the corresponding associated ground truth spike rate, ΔF/F signal, spike rate inferred by the default CASCADE model, spike rate inferred from the retrained CASCADE model, and spike rate inferred by the OASIS algorithm. Bottom row: Same as in top row panels but averaged across events, with absolute values indicated if possible. Prolonged spike rate is seen for the default CASCADE and OASIS models. The median number of spikes during events (1-s window around event) for ground truth vs. default CASCADE vs. retrained CASCADE is 17.2 vs. 10.0 vs. 11.8 spikes. Download Figure 5-1, TIF file. [file jneuro-45-e1187242025-s007.tif]
